# Supplementary material for: Co-modulation of clinical courses of hypertension and depression in public health facilities in Ethiopia: A six-month follow-up study protocol
Source: PLoS One. 2026 Feb 25;21(2):e0343829. doi: 10.1371/journal.pone.0343829 (PMC12935208; doi:10.1371/journal.pone.0343829)
Supplement: Attachment — Submitted filename: Response to technical review.docx [file pone.0343829.s001.docx]

1. Please complete all items on the Human Participants Research Checklist that are relevant for your submission.

- ***The Human Participants Research Checklist is completed and uploaded as a file type “Other”***

2. We note that you have indicated that you received funding for your study. If you received peer reviewed external funding, your submission may be considered for expedited editorial assessment. To be considered for expedited review, please upload the Funding letter you received, and English translation, as an ‘Other’ file. Please also provide proof that the funder administered independent peer review of your protocol. Examples of evidence include: a link to a description of the funder’s peer-review process; a letter from the funding agency confirming the independent peer review process; a list of the names and credentials of the peer reviewers (if available).

- ***The funding for this study was obtained from my own university. The funding is not external.***

3. Please ensure that your Methods section reports the status and timeline of the study, including an estimate of when A) participant recruitment will be completed (if applicable), B) data collection will be completed and C) results are expected. If any of these stages have already been completed, this should be indicated in the Methods section.

- ***Participant recruitment has not commenced yet. We planned to start the recruitment and data collection after the second week of September 2025. We expect to have the baseline data in January 2026. Data collection is expected to be completed by the end of June 2026. We anticipate having the study results by August 2026.***

4. We note you did not include a copy of the original, (signed or stamped) dated IRB/research ethics committee approval letter or waiver for all study sites as an ‘Other’ file.

- ***Ethical approval was obtained from the Institutional Review Board (IRB) of the College of Medicine and Health Sciences, Bahir Dar University, on April 2, 2025 (Protocol number: 3095/2025). And the approval letter is uploaded in the recommended file type (other)***
