# Supplementary material for: Co-modulation of clinical courses of hypertension and depression in public health facilities in Ethiopia: A six-month follow-up study protocol
Source: PLoS One. 2026 Feb 25;21(2):e0343829. doi: 10.1371/journal.pone.0343829 (PMC12935208; doi:10.1371/journal.pone.0343829)
Supplement: Attachment — Submitted filename: Response to Reviewers.docx [file pone.0343829.s002.docx]

Point-by-point response

|  | Comment /Questions | Responses |
| --- | --- | --- |
| Academic Editor | 1. Please ensure that your manuscript meets PLOS ONE's style requirements, including those for file naming. | Thank you for your time and consideration. We double-checked that the manuscript meets PLOS ONE's style requirements |
|  | 2. Thank you for stating the following financial disclosure: “This research is funded by Bahir Dar University under the BDU-LCM study grant number BDU-CMHS-005”  Please state what role the funders took in the study. If the funders had no role, please state: "The funders had no role in study design, data collection and analysis, decision to publish, or preparation of the manuscript." If this statement is not correct you must amend it as needed. Please include this amended Role of Funder statement in your cover letter; we will change the online submission form on your behalf. | Thank you so much. The Bahir Dar University has no role in the study. We want to state the following **in the financial disclosure**:  “The funders had no role in study design, data collection and analysis, decision to publish, or preparation of the manuscript.”  It can be revised as:  “This research is funded by Bahir Dar University under the BDU-LCM study grant number BDU-CMHS-005. The funders had no role in study design, data collection and analysis, decision to publish, or preparation of the manuscript.” |
|  | 3. Your ethics statement should only appear in the Methods section of your manuscript. If your ethics statement is written in any section besides the Methods, please move it to the Methods section and delete it from any other section. Please ensure that your ethics statement is included in your manuscript, as the ethics statement entered into the online submission form will not be published alongside your manuscript. | The ethics statement appears in the manuscript, and it is only in the methods section. |
|  | 4. If the reviewer comments include a recommendation to cite specific previously published works, please review and evaluate these publications to determine whether they are relevant and should be cited. There is no requirement to cite these works unless the editor has indicated otherwise | The reviewer’s comments do not include a recommendation to cite specific previously published works. |
|  | 5. Please review your reference list to ensure that it is complete and correct. If you have cited papers that have been retracted, please include the rationale for doing so in the manuscript text, or remove these references and replace them with relevant current references. Any changes to the reference list should be mentioned in the rebuttal letter that accompanies your revised manuscript. If you need to cite a retracted article, indicate the article’s retracted status in the References list and also include a citation and full reference for the retraction notice. | We have reviewed our reference list and are confident it is complete and correct. We have checked all the references in the Watch Database and PubMed metadata for retraction; none of the references in the list were retracted as of 18 January 2026. |
| Reviewer #1 | I want to congratulate the researchers for their planning. To increase the quality of the protocol, I have the following concerns.   1. In recruitment and data collection on page 7, line 81 & 82 “the study participants 82 who meet the inclusion criteria consecutively until a total of 485 participants (122 cases) is reached”. Here, your sample size is 485 (122 cases), which is manageable and it is expected that there will be huge number of such study participants in Bahir Dar city. If so, why the researchers planned and preferred to use “consecutively” than any random methods? Alternatively, why not systematic random sampling method? | Thank you so much for your encouragement and for taking the time to provide constructive feedback. This will definitely help us improve the quality of our protocol.   1. We planned to use consecutive sampling because we assessed the flow of cases in Bahir Dar and found it low or very low across facilities. This was observed because we are recruiting only newly diagnosed people with hypertension, excluding patients who plan to be transferred out of Bahir Dar city. On top of that, consecutive sampling is claimed to be reasonably representative when: a) multiple sites are included, b) all eligible participants are invited to participate, and c) the data collection period is long enough to include seasons (Mathieson 2014), which is the case in our study. In the manuscript, we have clarified in the Methods section, Sampling technique subsection (page 9, Line # 111), which reads as :   “ We will use consecutive sampling, where participants will be included in the study as long as they fulfil the inclusion and exclusion criteria until the targeted sample size is reached. Consecutive sampling technique is a common practice in healthcare research, particularly when the study population is infinite. It is claimed to be reasonably representative when: a) multiple sites are included, b) all eligible participants are invited to participate, and c) the data collection period is long. The participants of this study will be recruited from seven centres over ten months.”   2. On the other hand, other sampling approaches, such as systematic random sampling, cannot be feasible because they extensively increase the recruitment duration, making the study too costly. |
|  | 2. The researchers planned to include newly diagnosed adult hypertension patients and evaluate after 3 & 6 months. Is depression detectable at 3 month duration of hypertension? Will the level of co-morbidity be significant at this level? To become with best research finding, better to extend the study duration and the evaluation months as depression takes time. | Thank you again. We planned repeated measurements at 3 and 6 months for the following reasons: Conceptually, three months is an adequate time to observe incident cases of depression. PHQ-9 captures depressive symptoms over the previous two weeks (half a month). In a similar study conducted by our research team members using the PHQ-9, the incidence of depression was detected at two months in TB patients, and it was even higher at two months than at six months(Ambaw, Mayston et al. 2020). While a longer-duration study is clearly more advantageous than a shorter one, the cost and observability of comodulation at six months were the basis for determining the study's duration. |
|  | 3. The inclusion criteria focused on newly diagnosed adult hypertension patients. However, your consideration under is sample size is with depression 485 (363 without depression and 122 with depression). This raises a question “can we gain depression cases from newly diagnosed hypertensive patients?” Please, consider this. | In our study, we will include newly diagnosed adult patients with hypertension. All eligible participants will be screened for depression with the PHQ-9 at baseline. Because the prevalence of common mental illnesses in the general adult population in Ethiopia, from a systematic review (Kassa and Abajobir 2018), is 21.6%, we anticipate that at least 25% of the newly diagnosed hypertension patients (about 122 participants) would have depression on the PHQ-9. |

Ambaw, F., R. Mayston, C. Hanlon and A. Alem (2020). "Incidence of depression in people with newly diagnosed tuberculosis in Ethiopia: a cohort study." Global Mental Health **7**: e1.

Kassa, G. M. and A. A. Abajobir (2018). "Prevalence of common mental illnesses in Ethiopia: A systematic review and meta-analysis." Neurology, Psychiatry and Brain Research **30**: 74–85.

Mathieson, K. (2014). "Making sense of biostatistics: Types of probability sampling." Journal of Clinical Research Best Practices **10**(9): 1–2.
